# Supplementary material for: Reducing mosquito-borne disease transmission to humans: A systematic review of cluster randomised controlled studies that assess interventions other than non-targeted insecticide
Source: PLoS Negl Trop Dis. 2021 Jul 29;15(7):e0009601. doi: 10.1371/journal.pntd.0009601 (PMC8354450; doi:10.1371/journal.pntd.0009601)
Supplement: S2 Appendix — (DOCX) [file pntd.0009601.s002.docx]

**Appendix 2**

**Data fields**

1. Citation
2. Disease of interest
3. Inclusion/exclusion criteria
4. Setting
5. Study period
6. Primary outcome and data collection method
7. Secondary outcome and data collection method/s
8. Vector type/species
9. Spatial distribution of clusters
10. Method of randomization
11. Level of clustering (household, village, region etc)
12. Intervention/s used for mosquito control
13. Data analysis method
14. Qualitative results
15. Cohort age in years
16. No. clusters
17. No. participants
18. No. mosquito-borne disease cases in the intervention wing
19. No. mosquito-borne disease cases in the control wing
20. Loss to follow up
21. Effectiveness measures
22. Assessment of bias
23. Stated limitations
24. Was a difference in outcome found (if not was this attributed to the study design)?
25. Other comments
