# Supplementary material for: Reducing mosquito-borne disease transmission to humans: A systematic review of cluster randomised controlled studies that assess interventions other than non-targeted insecticide
Source: PLoS Negl Trop Dis. 2021 Jul 29;15(7):e0009601. doi: 10.1371/journal.pntd.0009601 (PMC8354450; doi:10.1371/journal.pntd.0009601)
Supplement: Attachment — Submitted filename: cover.pdf [file pntd.0009601.s006.pdf]

17 June 2021

Dear editors,

Thank you for your recommendations for our manuscript (ID: PNTD-D-21-00154R2).

1. I have reminded Prof. Stinear to confirm his authorship.
2. I have removed files which do not relate to the current submission.
3. The incorrect funding statement has been removed. No specific funding was received for this work.
4. The typo in the manuscript has been corrected to read 'Table 2'.
5. A full list of legends for the supporting information has been added to the manuscript.

Please let me know if there is anything else I can do to help at this stage.

Kind regards,

Jane Oliver
Lead author and Corresponding author

Postdoctoral Fellow
Department of Infectious Diseases
Melbourne Medical School
Faculty of Medicine, Dentistry and Health Sciences
University of Melbourne, Victoria, Australia 3010

Phone: +61 426 806 177 | Email: jane.oliver@unimelb.edu.au
