# Supplementary material for: Reducing mosquito-borne disease transmission to humans: A systematic review of cluster randomised controlled studies that assess interventions other than non-targeted insecticide
Source: PLoS Negl Trop Dis. 2021 Jul 29;15(7):e0009601. doi: 10.1371/journal.pntd.0009601 (PMC8354450; doi:10.1371/journal.pntd.0009601)
Supplement: Attachment — Submitted filename: Response to reviewers.docx [file pntd.0009601.s005.docx]

We would like to thank the peer reviewers and editors at *PLOS Neglected Tropical Diseases* for the time they have spent considering our article. We are grateful for their thoughtful comments and suggestions which we feel enrich the quality of our manuscript. We have responded to each of the comments as detailed in the tables that follow. Changes are marked using track changes in the revised manuscript.

**Reviewer 1**

| **Comment** | **Response** | **Location of change in manuscript** |
| --- | --- | --- |
| The authors have included 2 studies where non-targeted insecticide was applied in both arms. | There are three included studies where non-targeted insecticide was applied in both the control and the intervention arms (Andersson 2015, Yapabandra 2004, Hill 2007).  This inclusion is scientifically valid. These studies meet our inclusion criteria as these applications were separate from the study intervention. Such activities represent important sources of potential contamination bias, which researchers planning to conduct a cRCT should consider.  Greater emphasis on this point and more clarity around the justification for including such studies has been added to this review. | Title  Methods, page 6 paragraph 1.  Results, page 13, paragraph 2.  Table 2 – under ‘Yapabandara AM. 2004’.  Discussion, page 19, paragraph 2. |
| It is not clear that the included studies that "interrupt" transmission - I suggest "reduce". | Thank you for this suggestion. To avoid overstating the effects, "interrupt" has been replaced with “reduce” in the title. | Title. |
| Also this review has been conducted to inform the use of a cRCT to reduce mosquito borne transmission of Mycobacterium ulcerans, the causal agent of Bureli ulcer. "A novel intervention study for Buruli ulcer targeting mosquito vectors was proposed for a Buruli ulcer-endemic area of Victoria, Australia"  Thus a more infomative title for this article:  Cluster randomised controlled studies to reduce mosquito-borne disease transmission to humans. A systematic review in the context of Bureli ulcer transmission. | We have not conducted this review in the context of Buruli ulcer transmission, but rather in the context of reducing mosquito-borne diseases generally; hence our inclusion of studies with various species of mosquito. For this reason we do not feel the proposed title is entirely suitable for this manuscript, but have made other edits in line with the recommendation. |  |
| Support for the evidence that mosquitoes transmit Bureli ulcer, appears to be weak.  Page 3, second paragraph "Mycobacterium ulcerans is considered an environmental pathogen…., evidence implicates biting insects as having a key role in the transmission of M. ulcerans. [16, 17]."  Thus in their proposed intervention it is important that the methods used to reduce mosquitoes density are highly efficacious. | There is growing evidence that mosquitoes transmit Buruli ulcer in some endemic areas. The description of the evidence for mosquito transmission has been strengthened.  In the Discussion, we have noted the importance of having a highly efficacious mosquito control intervention in order to disrupt transmission. | Introduction, page 3 paragraph 2.  Discussion, page 18 paragraph 1. |
| Abstract: State the date range applied to interrogate the listed medical research databases. | Added | Abstract |
| Abstract: Needs more care with English, for example in the abstract, results section: "Eight eligible cRCTs were conducted between 1994-2013 in variable settings in the Americas and Asia were identified." Suggestion "Eight eligible cRCTs conducted between 1994-2013 were identified in a variety of settings in the Americas and Asia. | Correction made.  The manuscript has been proof-read with reference to this comment. | Abstract |
| Abstract: The term "Source reduction" requires definition. | Edited for clarity | Abstract and Author summary. |
| Page 6  Data abstraction: The full …….disease and vector; study setting and period;. Please change "period" to "date" | Edited | Methods, page 6, paragraph 3 |
| The aim was to review cRCT designs used to investigate interventions without non-targeted insecticide for reducing mosquito-borne disease transmission to humans, and comment on the  strengths and weaknesses of these study designs. They have included articles with non-targeted insecticide when applied in control and intervention groups. This needs to be justified. | Yes – please see the response to the first comment. |  |
| DISCUSSION - first sentence:  "This review is unique in that it considers the effects of mosquito control interventions on associated disease, rather than on entomological indicators only"  Please clarify. Many studies and reviews consider the effect of mosquito control interventions on associated disease - Anopheline studies and malaria and many others.  The novel nature of the proposed Buruli ulcer prevention strategy is, I believe, that mosquitoes have not previously been targeted as a means of reducing this disease. | We acknowledge this point and have removed the first sentence. | Discussion, Page 18, paragraph 1 |

**Reviewer 2**

| **Comment** | **Response** | **Location of change in manuscript (Page, paragraph)** |
| --- | --- | --- |
| Well written paper. Considering that the review include small number of eligible studies, it would be helpful to discuss advantages and disadvantages of other types of study design used for similar studies | Thank you for this suggestion. Discussion around the advantages and disadvantages of other types of study design used for similar studies has been added. | Discussion, Page 18, paragraph 1. |
