# Supplementary material for: Reducing mosquito-borne disease transmission to humans: A systematic review of cluster randomised controlled studies that assess interventions other than non-targeted insecticide
Source: PLoS Negl Trop Dis. 2021 Jul 29;15(7):e0009601. doi: 10.1371/journal.pntd.0009601 (PMC8354450; doi:10.1371/journal.pntd.0009601)
Supplement: Attachment — Submitted filename: R2 Response to Reviewer Comments-7Jun.docx [file pntd.0009601.s007.docx]

| 21 June 2021  The authors would like to thank the reviewer and the editors for their thoughtful suggestions and for the time they have spent considering our manuscript.  We are pleased to make many of the changes which they suggested. We feel that these changes enrich the quality of our manuscript.  In response to the editors’ comments  1. I have reminded Prof. Stinear to confirm his authorship.  2. I have removed files which do not relate to the current submission.  3. The incorrect funding statement has been removed. No specific funding was received for this work.  4. The typo in the manuscript has been corrected to read ‘Table 2’.  5. A full list of legends for the supporting information has been added to the manuscript.  The response to each of the reviewer’s suggestions is discussed in detail as follows. | | |
| --- | --- | --- |
| **Comment** | **Response** | **Location of change in manuscript** |
| Strictly limited details of the planned Buruli ulcer cRCT add interest to this article but should be succinct. Similar considerations apply to the Discussion.  The article thus requires major revision. | We have reviewed and revised the Introduction and Discussion sections with this point in mind. | - |
| The English also needs revision. | The authors are all native English speakers and we are not sure what the issue is, however we have reviewed the manuscript considering this point. We note that a previous reviewer described the article as well-written. | - |
| In addition, given that only three articles have been identified by this systematic review, perhaps the article would be more suitable as a short communication. | We feel that the current format enables the finding to be discussed in enough detail to properly contextualise the findings. | - |
| The phrase "interventions with targeted insecticide" is considerably clearer than "interventions without nontargeted insecticide". | This is true, but the meaning differs.  We have replaced “interventions without nontargeted insecticide” with the phrase  “interventions other than non-targeted insecticide” throughout the document to enhance clarity. | Throughout document |
| ***Author summary*** | | |
| Point 2 This literature review identified three studies with an intervention which did not include non-targeted use of insecticide and was associated with statistically significant reductions in the disease of interest and in entomological indicators.  Rewrite: This literature review identified three intervention studies, without non-targeted use of insecticide, that were associated with statistically significant reductions. | Re-written as suggested, however we retained the description of what outcome measures these reductions were noted for. | Author summary, point 2. |
| Point 3 & 4 are repetitive and should be combined. | Combined as suggested | Author summary, point 3. |
| Point 5 Is the final statement supported by the evidence reviewed? "Regular administration of larvicide to potential breeding sites that are unsuitable for source reduction may supplement this intervention strategy." If not, I think it should be removed. | Yes, we believe this point is supported by the evidence. Two included studies reported that larvicide in breeding sources was effective for *Anopheles* control. While this is a different mosquito species to *Aedes,* there is no reason to suspect this finding would not hold relevance to *Aedes* control. Furthermore, the effective intervention in the third included paper included placing small fish in drinking water sources in Mexico. While this is not larvicide, it may have had a similar effect by reducing the ability to mosquitoes to breed in water sources. | - |
| Full Title: "Reducing mosquito-borne disease transmission to humans: A systematic review of cluster randomised controlled studies that assess interventions other than non-targeted insecticide."  Suggest: "Reducing mosquito-borne disease transmission to humans: A systematic review of cluster randomised controlled studies that assess interventions using targeted insecticide."  Short title: Intervention studies without non-targeted insecticide to reduce mosquito-borne disease.  Suggest: "cRCT using targeted insecticide to reduce mosquito-borne disease." | We have kept the original title as we did not restrict to studies that included targeted insecticide.  The short title has been modified for consistency. | Short title |
| ***Introduction*** | | |
| Section on Buruli ulcer only need mention: "This literature review was developed to inform the design of a future cluster randomised control study (cRCT) aiming to reduce Buruli ulcer transmission via a mosquito control intervention that does not include non-targeted insecticide spraying. The aim was to review cRCT designs used to investigate interventions without non-targeted insecticide for reducing mosquito-borne disease transmission to humans, and comment on the strengths and weaknesses of these study designs." | Edited for succinctness with most references to Buruli ulcer research articles removed. | Page 3, paragraph 2. |
| ***Methods*** | | |
| The authors definition of studies eligible for this review Page is suitable. The identification and selection of articles is well described, follows standard procedures, and covers "from the earliest available sources" up to July 2019. There are 8 articles included in the review but in the final analysis only three met their criteria. They quote a previous review of cRDT in this field (Alvarado-Castro V, *et al*. (2017), ref 41). It would be informative to include this in the introduction as an example of a review on entomological endpoints and highlight reasons for using disease endpoints. | Reference to the Alvarado-Castro V, *et al.* review has been made in the Introduction. | Page 3, paragraph 1. |
| The inclusion criteria for studies in the review needs clarification.    Page 6, top paragraph. This indicates that studies with non-targeted insecticide/s applied in both the control and intervention arms were included.  Page 6, last paragraph the Eligibility criteria states "Intervention targeted mosquitoes without non-targeted use of insecticide." | Inclusion criteria clarified for consistency. | Page 5, paragraph 3.  Pahe 6, paragraph 1. |
| Page 13, Contamination between clusters probably occurred in several of the studies and are listed in Table 2. These are real world results that can inform future studies and this is a strength of this article. | Comment made | Page 18 paragraph 1 |
| ***Results*** | | |
| Page 8, top  Clarify the meaning of "title/abstract" as  Title and abstract, Title or abstract etc | Clarified | Page 8, paragraph 1. |
| Page 8, top  Following searches of the five medical research databases, 1,471 article citations were identified of which 391 articles underwent title/abstract screening (with 1,080 citations identified as duplicates and discarded; Figure 1). Rewrite: Following searches of the five medical research databases, 1,471 article citations were identified of which 1080 were duplicates and 391 articles underwent title/abstract screening (Figure 1). | Clarified | Page 8, paragraph 1. |
| ***Discussion*** | | |
| As mentioned previously, strictly limited / succinct details of the planned Buruli ulcer cRCT add interest to this article. The Discussion needs to discuss the results of the literature review with minor mention of the Buruli ulcer cRCT. It therefore needs rewriting. | The Discussion has been edited for succinctness in regards to the planned Buruli ulcer cRCT.  Note that several comments have been retained as these hold relevance to intervention studies for mosquito-borne disease control generally. Edits have been made to emphasis this relevance. | Page 18, paragraph 1.  Page 20, paragraph 2.  Page 21, paragraph 1. |
